# Hepatitis E Virus Cysteine Protease Has Papain Like Properties Validated by *in silico* Modeling and Cell-Free Inhibition Assays

**DOI:** 10.3389/fcimb.2019.00478

**Published:** 2020-01-23

**Authors:** Shweta Saraswat, Meenakshi Chaudhary, Deepak Sehgal

**Affiliations:** Virology Lab, Department of Life Sciences, Shiv Nadar University, Greater Noida, India

**Keywords:** HEV-protease, papain like enzyme, baculovirus expression system, *in silico* modeling, biochemical characterization

## Abstract

Hepatitis E virus (HEV) has emerged as a global health concern during the last decade. In spite of a high mortality rate in pregnant women with fulminant hepatitis, no antiviral drugs or licensed vaccine is available in India. HEV-protease is a pivotal enzyme responsible for ORF1 polyprotein processing leading to cleavage of the non-structural enzymes involved in virus replication. HEV-protease region encoding 432–592 amino acids of Genotype-1 was amplified, expressed in Sf21 cells and purified in its native form. The recombinant enzyme was biochemically characterized using SDS-PAGE, Western blotting and Immunofluorescence. The enzyme activity and the inhibition studies were conducted using Zymography, FTC-casein based protease assay and ORF1 polyprotein digestion. To conduct ORF1 digestion assay, the polyprotein, natural substrate of HEV-protease, was expressed in *E. coli* and purified. Cleavage of 186 kDa ORF1 polyprotein by the recombinant HEV-protease lead to appearance of non-structural proteins viz. Methyltransferase, Protease, Helicase and RNA dependent RNA polymerase which were confirmed through immunoblotting using antibodies generated against specific epitopes of the enzymes. FTC-casein substrate was used for kinetic studies to determine Km and Vmax of the enzyme and also the effect of different metal ions and other protease inhibitors. A 95% inhibition was observed with E-64 which was validated through *in silico* analysis. The correlation coefficient between inhibition and docking score of Inhibitors was found to have a significant value of *r*^2^ = 0.75. The predicted 3D model showed two domain architecture structures similar to Papain like cysteine protease though they differed in arrangements of alpha helices and beta sheets. Hence, we propose that HEV-protease has characteristics of “Papain-like cysteine protease,” as determined through structural homology, active site residues and class-specific inhibition. However, conclusive nature of the enzyme remains to be established.

## Introduction

Hepatitis E virus (HEV) is one of the most important viruses responsible for water born epidemics (Kamar et al., 2014). It is primarily transmitted through the faeco-oral contaminated drinking water. HEV was discovered in 1983 in an outbreak of unexplained hepatitis in Soviet soldiers in Afghanistan. Although, HEV is more prevalent in developing countries due to poor sanitation and water supplies (Cao and Meng, 2012) however, cases of HEV infection in industrialized countries like Europe, USA and Japan are becoming more common (Minuk et al., 2007; Bendall et al., 2008; Mushahwar, 2008). HEV causes self-limiting acute infection in approximately 20 million people annually, with a global mortality rate of 3% (Jameel, 1999; Nan and Zhang, 2016). This mortality rate remarkably increases up to 30% in the infected pregnant women in their third trimester due to fulminant liver failure (Navaneethan et al., 2008; Aggarwal and Naik, 2009). Infection with HEV represents an important global public health problem due to significant morbidity and mortality (Gupta and Agarwala, 2018). Currently a vaccine has been developed but licensed only in China, thus there is no vaccine or therapeutics available against HEV infection elsewhere. Also, there is no accepted treatment for HEV but the treatments of both interferon and/or ribavirin as a combinatorial therapy have been used successfully to treat chronic HEV infection (Kamar et al., 2014), though it has some side effects.

Genetically, HEV genome is a non-enveloped single-stranded positive sense RNA of ~7.2 kb long and contains three partially overlapping open reading frames ORF1, ORF2, and ORF3 (Tam et al., 1991; Tsarev et al., 1992; Ahmad et al., 2011). HEV ORF3 translates into a small phospho-protein that modulates some of the host-regulatory functions including establishment of infection and virion egress (Graff et al., 2005; Chandra et al., 2008; Yamada et al., 2009). ORF2 forms a 660 amino acid (72 kDa) protein and its processed form constitutes the viral capsid. ORF1 is the largest ORF, 5,109 bases long and translated into 1,693 amino acids, which encode the non-structural polyprotein of ~186 kDa, essential for viral replication (Ansari et al., 2000). Computational analysis of ORF1 has identified seven putative domains (Koonin et al., 1992). These include an active methyltransferase domain (Met), Y domain (Y) (Parvez, 2017), papain-like cysteine protease (PCP) (Parvez, 2013; Paliwal et al., 2014), a proline -rich region that contains a hypervariable region (H), X -domain (X), helicase (Hel), and an RNA dependent RNA polymerase (RdRP) From N- to C-terminal (Koonin et al., 1992; Parvez, 2017). Except PCP and Y domain, all other putative domains have been partially characterized and their functions have been predicted bioinformatically and some of them even experimentally (Agrawal et al., 2001; Magden et al., 2001; Karpe and Lole, 2010a,b). A recent report has identified an additional ORF4 in genotype-1 HEV, which is presumed to play an essential role in viral replication (Nair et al., 2016).

Various attempts have been made to study ORF1 processing and validate proteolytic activity of the PCP domain but not much success has been achieved. Expression of ORF1 in cell free system and the bacteria showed a 186 kDa polyprotein (Ansari et al., 2000) while the same construct, expressed using vaccinia virus showed two fragments of 107 kDa and 78 kDa in HepG2 cell (Ropp et al., 2000). In another study, transfection of infectious HEV RNA into HepG2 cells showed processed ORF1 fragments, of 35, 38, and 36 kDa using anti-MetT, anti-Hel and anti-RdRp antibodies, respectively (Panda et al., 2000). Similarly expression of ORF1 using baculovirus system showed processing of ORF1 as eight fragments that was inhibited by cell permeable cysteine protease inhibitor (E-64d) (Sehgal et al., 2006) but this could not be concluded whether the ORF1 processing was due to HEV-protease or an host-encoded protease. In recent report the role of host factor Xa and thrombin was postulated to initiate the HEV ORF1 processing but it still needs further validation (Kanade et al., 2018). Recently, mutation in conserved cysteine and histidine residues in the putative protease inhibits its proteolytic activity indicating its role in ORF1 processing and viral replication (Paliwal et al., 2014).

The HEV-protease has been reported to act as papain-like cysteine proteases with conserved catalytic dyad (Cys and His) (Koonin et al., 1992; Parvez and Khan, 2014), but it was also reported as a chymotrypsin like protease in another study (Paliwal et al., 2014). The main goal of our study has been to validate the nature of HEV-protease and established its role in the polyprotein processing. Since, other viral proteases have been identified as a potent antiviral target in literature (Lv et al., 2015) hence, HEV-prorease was thought to be a putative inhibitor for HEV replication. Therefore, we report the expression of a soluble, catalytically active recombinant HEV protease using baculovirus system. Further, we developed highly sensitive and reliable cell free assays to screen the cysteine protease activity. Also a 3D model of the HEV protease was generated to identify possible active sites and the residues responsible for binding of inhibitors. We considered the HEV-protease to be a Papain like cysteine protease. Collectively, this study significantly advances our understanding of the structure and function of HEV protease.

## Result

### Generation of Recombinant Baculovirus Containing HEV-Protease

**Figure 1:**
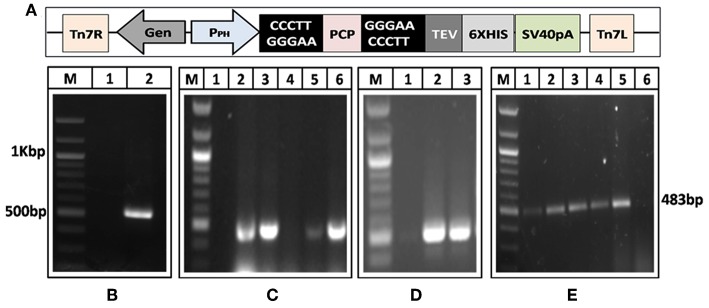
Generation of recombinant baculovirus to express HEV-protease. **(A)** Amplification of HEV-protease gene from pSK-HEV2 plasmid; lane M, 100 bp DNA ladder, lane 1, negative control (non-template control); lane 2, PCR amplified HEV-protease fragment. **(B)** Vector map of recombinant pFastBac-PCP showing integration of PCP gene under PH promoter. **(C)** Colony PCR of DH5α transformants to identify clones having PfastBac-PCP construct; lane M, marker, lane 1 negative control, lane 2–5 amplification from different colonies obtained after transformation of PfastBac-PCP construct in DH5α cells, lane 6, Positive control (amplified product of pSK-HEV2 plasmid). **(D)** Analysis of recombinat bacmid DNA by PCR; lane M, marker, 1, negative control (amplification without template), lane 2, amplification from recombinant bacmid containing HEV-protease, lane 3, Positive control (amplified product of pSK-HEV2 plasmid), **(E)** Production of recombinant baculovirus in transfected Sf21 cells; lane M, marker, lane 1–4, amplification from recombinant baculovirus infected Sf21 cells. lane 5, Positive control (PCR amplification from pSK-HEV2 plasmid); lane 6, Amplification from uninfected Sf21 cells.

A 482 bp segment encoding HEV-protease was amplified using pSK-HEV-2 clone as template by PCR (Figure 1A). The purified HEV-protease amplicon was cloned in pFastBac/CT-TOPO vector in frame with 6xHis at C-terminal, under polyhedrin (PH) promoter (Figure 1B). Screening of selected clones by PCR using gene specific primers confirmed integration of desired sequence (Figure 1C). Further, DNA sequencing confirmed the clone in correct reading frame with 100% similarity to the PCP sequences of the genotype-1 of HEV (Supplementary Figure 1, Data Sheets 2, 3). Transformation of pFastBac-PCP in DH10Bac cells resulted in the formation of recombinant bacmid carrying HEV-protease sequence under the PH promoter and the recombination was confirmed through PCR (Figure 1D). Hence, a recombinant baculovirus containing the HEV-protease expression cassette was formed by transfecting the recombinant bacmid into Sf21 insect cells. Transfection leads to cell enlargement, granulation, and vacuole formation after 72 h. Integration of HEV-protease was confirmed by PCR from baculovirus genomic (Figure 1E).

### Confirmation of HEV-Protease Expression

**Figure 2:**
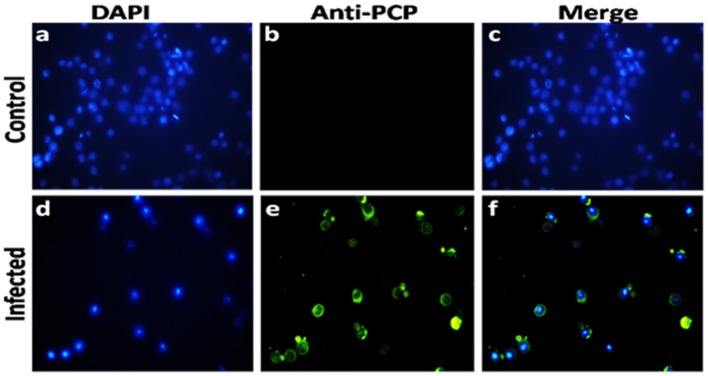
Immunofluorescence analysis of HEV-protease expression in Sf-21 cells. **(a,d)** represent the DAPI-stained uninfected and infected sf21 cells; **(b,e)** represent immunoflorescence of uninfected and infected Sf21 cells after staining with HEV-protease epitope specific primary antibody and goat anti-rabbit IgG-Alexa Fluor 488 secondary antibody; **(c,f)** shows composite image of DAPI- and Alexa Fluor 488-stained uninfected and infected Sf21cells.

In order to confirm the expression of HEV-protease in infected Sf21 cells, an indirect immunofluorescence assay was performed using HEV-protease epitope specific antibody. The Baculovirus-infected Sf21 cells produced fluorescence after staining with HEV-protease antibody (Table 1, Figure 2).

**Table 1:** Peptide sequence of antibodies generated against epitope of all non-structural proteins of HEV genotype-1.

**S.no**.	**Protein name**	**Peptide sequence**	**Location on ORF1**
1	Methyl transferase	AGRDVQRWYTAPTRC	111–124
2	HEV-protease	LDPRVLVFDESAPC	444–457
3	Helicase	TADARGLIQSSRAH	1,505–1,518
4	RNA dependent RNA polymerase	PKESLKGFWKKHSG	1,156–1,169

### Expression and Purification of HEV-Protease

**Figure 3:**
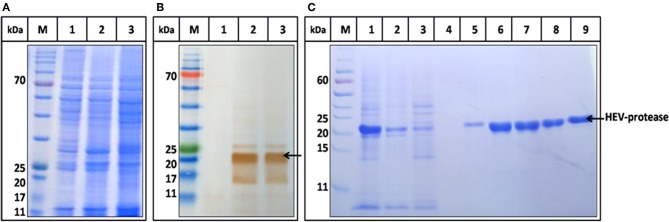
Expression and Purification profile of HEV-protease. Highest expression level was seen at 48 h of post infection at 10 MOI. **(A)** SDS-PAGE and coomassie blue staining of Sf21 expressed HEV-protease. **(B)** Western blot analysis of HEV-protease by HEV PCP antibody; lane M marker, lane 1 cell lysate of uninfected Sf21 cells, lane 2 and 3, cell lysate of infected Sf21 cells after 48 and 72 h of post infection. **(C)** SDS-PAGE and coomassie blue staining of Ni-NTA chromatography fractions: lane M, marker; lane 1, solubilized protein; lane 2, Flow through; lane 3,4, Wash fractions; lane 5–9, Elution Fractions.

The highest expression of HEV-protease was obtained at 27°C at 10 MOI after 48 h of infection. SDS-PAGE analysis showed expression of ~18 kDa HEV-protease on SDS-PAGE (Figure 3A) which was confirmed by immunoblotting using anti HEV-protease antibody (Figure 3B). The expressed protein was solubilized using 1% CHAPS and 10% DMSO. Purification was performed by Ni^2+^-NTA affinity chromatography under native conditions. The eluted protein was >95% pure by gel analysis (Figure 3C).

### Substrate Specific Catalytic Activity of HEV Protease

**Figure 4:**
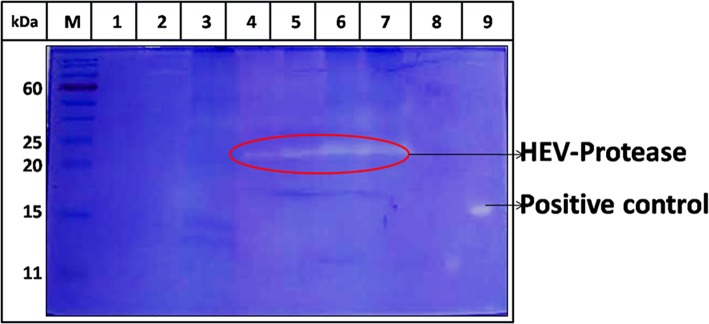
Zymography of HEV protease using gelatin as a substrate. Purified HEV-protease was loaded in the wells for determining cleavage of gelatin; lane M, protein ladder, lanes 1 and 2 were loaded with 10 and 20 μl of solubilization buffer to eliminate any contaminating protease activity, lane 3 was loaded with 20 μl cell sup of uninfected Sf21 cells, lane 4–7 were loaded with increased quantity of HEV-protease (5,10,15, and 20 ng, respectively), lane 8 was left blank, and lane 9 was loaded with 5 ng of trypsin as a positive control. The cleavage of gelatin confirmed the protease activity. There was no digestion of gelatin in the negative control lane, indicating the absence of any non-specific protease activity.

The activity of purified HEV-protease was determined by negative staining using native gelatin zymography. Gelatin is a non-specific substrate for protease and forms a clear zone on zymography assay due to its digestion. Increasing quantity of HEV-protease (5, 10, 15, and 20 ng) resulted with increased intensity of clear zone due to digestion of gelatin (Figure 4, lane 4–7). Digestion of gelatin was not seen in negative control (Figure 4, lane 2,3), indicating specificity of the assay.

**Figure 5:**
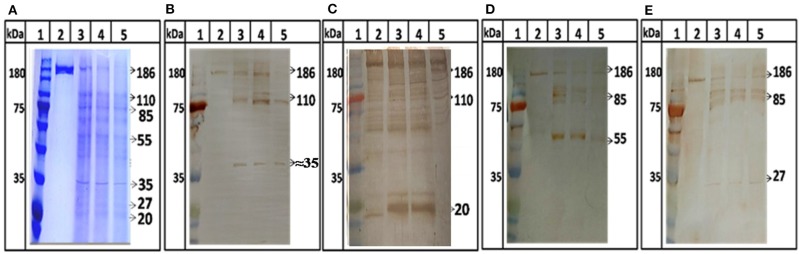
ORF1 digestion by HEV Protease. Full length ORF1 protein of HEV was purified and used as the natural substrate of HEV-protease. The ORF1 protein was digested with HEV-protease and fractionated on SDS-PAGE. Western blot was performed using antibodies generated against other domains of ORF1 polyprotein. **(A)** SDS-PAGE and coomassie blue staining of digested ORF1 using protease. lane 1, Marker; lane 2, Undigested ORF1; lane 3–5, digested ORF1 after 1, 3, and 5 h digestion. **(B)** western blot analysis using anti-Met antibodies, the Met was seen as the digested product of ORF1 (**B**, lane 3–5). Similarly, the blots were probed with protease, RdRp and helicase antibodies **(C–E)**. Lane 2 represents undigested ORF1 while lane 3–5 represents the ORF1 digestion at different time points (1, 3, and 5 h respectively). The figure clearly showed 186 kDa ORF1 and the Met, RdRp and Hel as proteolytic fragment of ORF1.

Further, protease activity was confirmed by digestion assay using ORF1 as a substrate. ORF1 has been reported to cleave in putative domains of MeT, PCP, Hel, and RdRp (Sehgal et al., 2006). For the first time we showed digestion of ORF1 polyprotein into smaller fragments. After digestion, 186 kDa polyprotein breaks in to 110 and 85 kDa products which further breaks into smaller product of 55, 37, 27, and 18 kDa (Figure 5, lane 3–5). No autolysis has been seen in full length ORF1 even after 24 h of incubation at 37°C (Figure 5A, lane 2) validating the specificity of proteolysis of ORF1 by HEV-protease in digestion assay. To confirm the processing of ORF1, digested samples were immunoblotted using anti-MeT, anti-protease, anti-RdRp and anti-Hel antibodies (Table 1). Approximately 35, 20, 55, and 27 kDa size of bands were visualized with anti-Met, anti-protease, anti-RdRp and anti-Hel antibodies corresponding to the size of putative domain of methyletransferase (Figure 5B, lane 3–5), protease (Figure 5C, lane 3–5), RdRp (Figure 5D, lane 3–5), and Helicase (Figure 5E, lane 3–5). This suggests the role of HEV-protease in cleavage of ORF1 polyprotein.

### Enzyme Kinetics

**Figure 6:**
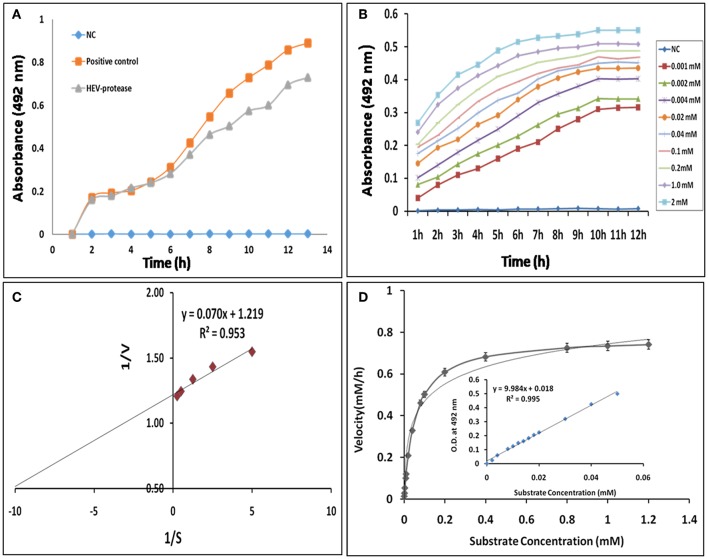
Enzyme kinetics of HEV protease: **(A)** FTC-casein based protease assay was developed for the determination of HEV protease activity. A negative control (NC) reaction was performed without enzyme, which did not show any change in the absorbance. **(B)** Real-time profile of proteolytic assay with increasing substrate concentrations, ranging from 0.01 to 2 mM, using 1 μM protease was performed and the absorbance measured at different time points every 1 h till 12 h till increase in absorbance was observed. Background absorbance (NC) was subtracted for clarity for comparison. **(C)** Lineweaver–Burk plot and regression equation plotted to determine km and Vmax of HEV-protease. **(D)** Reaction velocity (mM/h) calculated with the help of standard curve and plotted over different range of substrate concentration (means of three independent experiments performed in triplicate).

Next, FTC-casein was used as a substrate to determine the activity of HEV protease. The cleavage of FTC-casein results TCA-soluble, FTC-peptides in the presence of active protease. Protease activity was quantified by measuring absorbance at 492 nm. As seen in Figure 6A significant protease activity could be detected as compared to the mock reaction. Protease activity was proportional to the quantity of the substrate. Signal saturation was obtained at 9 h (Figure 6B). Further experiments were carried out to estimate kinetic parameters of HEV protease using the above conditions Reaction velocity V_0_ (mM/h) was found to be different for each substrate concentration S (mM). Accordingly, different V_0_ and S the Lineweaver-Burk graph was plotted against 1/V and 1/S to calculate Km and Vmax. Through the slope and the interception of the plot of 1/V vs. 1/S, the exact values of Km and Vmax were determined to be 0.054 mM and 0.77 mM/h, respectively (Figures 6C,D).

### Optimization of Reaction Conditions of Protease Activity

**Figure 7:**
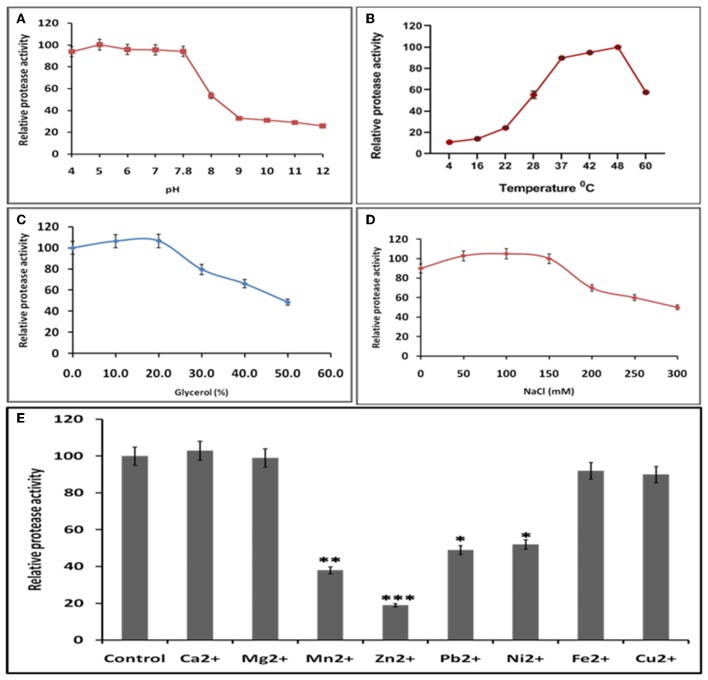
Optimal reaction conditions for the protease activity of HEV-protease **(A)** Effects of pH on protease activity. HEV protease and substrate were incubated with various buffers of pH ranging from 4 to 12. The relative activity was calculated at different pH at pH 7.8 as 100%. **(B)** Effects of temperature (4–60°C), the relative activity was calculated by taking activity at 37°C as 100%. **(C)** Effect of glycerol and **(D)** NaCl concentration on protease activity. HEV protease and substrate were incubated in a buffer of 50 mM Tris (pH 7.8) containing the indicated concentrations (0, 50, 100, 150, 200, 250, and 300 mM) of NaCl. **(E)** Effects of various divalent cations (2 mM) on protease activity (*p < 0.01, **p < 0.001, ***p < 0.0001) with respect to control.

Buffer and assay conditions were optimized to find activity at pH 4–7.8. Higher pH impaired the protease activity significantly in comparison to lower pH (Figure 7A). The optimal temperature for protease activity was found to be 37–48°C (Figure 7B). The effect of glycerol (anti-chemotropic agent) was also observed. At 10–20% glycerol concentration, slight increase in protease activity was seen. However, further increase in glycerol concentration resulted in the decrease of protease activity (Figure 7C). The results with different Na+ concentrations indicated that the optimal concentration was around 50–150 mM and that the enzyme is relatively insensitive to Na^+^ (Figure 7D) Analysis of the effect of different divalent cations like Ca^2+^, Cu^2+^, Mg^2+^, Mn^2+^, Ni^2+^, Zn^2+^, Fe^2+^, and Pb^2+^ on the activity of HEV protease revealed that Ca^2+^, Mg^2+^, Pb^2+^, and Fe^2+^ had no effect, however, Zn^2+^ (*p* = 0.0001), Ni^2+^, Pb^2+^ (*p* = 0.01) and Mn^2+^(*p* = 0.001) significantly decreased the protease activity (Figure 7E).

### Analysis of the Effect of Protease Inhibitors

**Figure 8:**
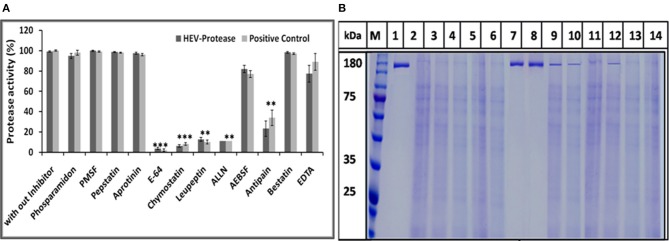
Inhibition assay: **(A)** Effects of different protease inhibitors on protease activity. The FTC-casein based protease assay was performed in presence of various inhibitors. The relative protease activity was determined by taking protease activity as 100% without inhibitor. The standard errors of the means of results from three independent experiments have been seen. The asterisk indicates statistical significance (**p < 0.001, ***p < 0.005) with respect to control (without inhibitors). **(B)** Activity of HEV protease was studied in the presence of twelve protease inhibitors. lanes 1 undigested ORF1, lane 2 digested ORF1 without any inhibitor. Lanes 3–14 represent ORF1 over digestion (5 h at 37°C) in the presence of 1× concentration of various inhibitors. By this time, active protease without any inhibitor (lane 2) had cleaved the entire pORF1. The most effective inhibition was observed with E-64 and chymostatin (lanes 7 and 8, respectively).

The effect of different protease inhibitors was tested using FTC-casein protease assay in the presence of inhibitors. The enzyme was completely resistant to serine proteases inhibitors (PMSF, AEBSF, aprotinin), aspartic proteases (pepstatin), amino-peptidases (bestatin), metallo-endoproteases (phosphoramidon). The enzyme was moderately inhibited by cysteine protease inhibitors (antipain, leupeptin, ALLN, chymostatin, and E-64) (Figure 8A). Minimum relative protease activity seen with E-64 suggested it to be a papain like cysteine protease. Further, activity of HEV protease on ORF1 digestion was tested in the presence of twelve different protease inhibitors in independent reactions (Figure 8B). It was observed that HEV protease was strongly inhibited by E-64 (99%) and chymostatin (98%). Moderate inhibition was seen with leupeptin, ALLN and antipain whereby no inhibition was observed with phosphoramidon, PMSF, AEBSF, aprotinin, pepstatin (Figure 8B).

### *In silico* Analysis of HEV-Protease

**Figure 9:**
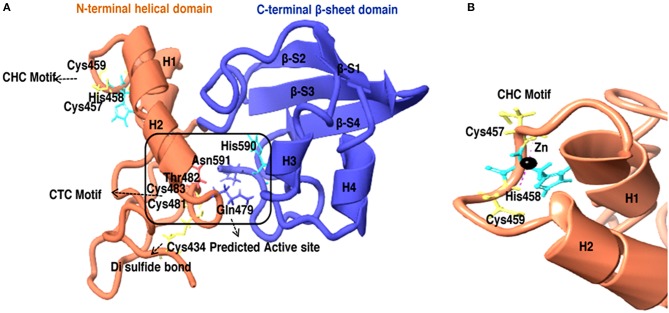
**(A)** 3D model of HEV-Protease found to be similar with Papain like cysteine protease as to have conserved catalytic triad and “papain-like β-barrel fold.” **(B)** Predicted Zinc coordination in the HEV PCP model, the diagrammatic representation of CHC motif and Zn binding site.

To further know the active site residue involved in the interaction between HEV-protease with inhibitors, its 3D structure has been modeled which also showed the nature of the HEV-protease. The predicted model showed two domains architecture, N-terminal helix and C-terminal β-sheet domain similar to papain-like cysteine proteases (Verma et al., 2016) but differ in arrangement of secondary structure elements. An active site (catalytic pocket) for substrate binding was present between two domains. The residues present in the predicted active site cleft were Gln479, Thr482, Cys483, Val507, Asp508, Leu582, Pro509, Ile561, Glu588, Arg589, His590, Asn591, Leu592, Met474, Lys457, Leu476, Phe575, and Gly478. A “Cysteine(Cys)-Histidine(His)-Asparagine(Asn)” catalytic triads has also been seen between N-terminal helical domain and a C-terminal β-sheet domain.Cys483-His590-Asn591 residues were found to be involved in this catalytic triad, which is the main characteristic of the “papain” family. One Di-sulfide bond observed between Cys434 and Cys481 necessary for stability and the proper folding of the protein. Residues present in CTC motif (Cys481 and Cys483) and CHC motif (Cys457 and Cys 459) may be involved in zinc metal ion coordination and catalysis (Figure 9A). It is reported that the Zn binding site present on the opposite side of active site (Herold et al., 1999), which even validated by crystal structure of hepatitis C virus NS3 proteases (Stempniak et al., 1997; Barbato et al., 1999; Arasappan et al., 2005) picornavirus 2A (Yu and Lloyd, 1992) PL1pro of HCoV-229E (Ziebuhr et al., 2007), p150 of RUBV (Zhou et al., 2007), and Lpro of FMDV (Guarne et al., 2000). The diagrammatic representation indicates presence of CHC motif, opposite to the predicted active site, could be zinc binding site of HEV-protease (Figure 9).

### Docking Study of Inhibitors for the Validation of HEV PCP Model

**Figure 10:**
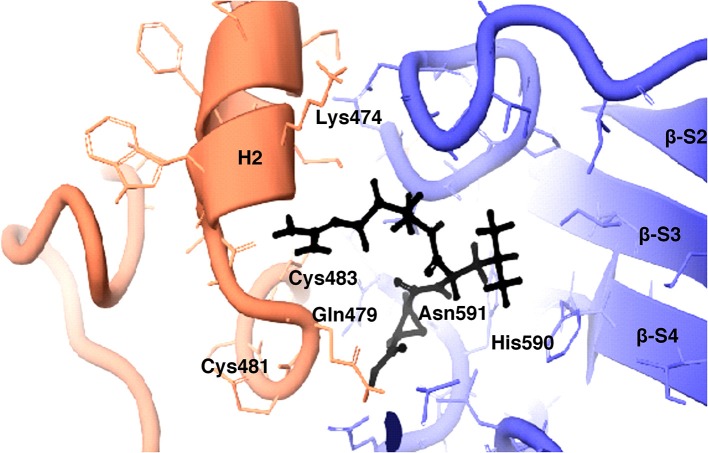
3D view of cysteine protease inhibitor E64 docked in the binding pocket of the HEV-protease model. Inhibitor E64 is shown as stick with color code pink. The H- bonds are highlighted by dotted yellow lines. Some part of the ribbon has been removed for clarity. Residues present in active site are shown as CPK in Gray color. Only catalytically important residues are marked. These residues are located in the substrate binding cleft.

The inhibition efficiency of all the inhibitors used in protease assay was also proved by docking studies to know the interaction between inhibitors and predicted active site residues of HEV-protease. Results summarized in Table 2 showed that most of the active compounds had good agreement between the docking score and experimental results (*r*^2^ ≈ 0.75). It suggests that the parameters of docking simulations and quality of structural model are good in reproducing experimental course of these compounds in the modeled HEV PCP. The observed difference among E64 docking scores and interacting residues may be due to difference in binding affinity of different inhibitors as observed in inhibitory efficiency. Four inhibitors (E64, Chymostatin, Leupeptin, and ALLN) showed good ligand–receptor interactions that correlated better with the enzyme inhibition activity of recombinant HEV PCP protein with Glide docking (Table 2). Docking result shows that cysteine protease inhibitors E64, Chymostatin, Leupeptin, and ALLN (docking score −6, −5.1, −5.8, −4.3 respectively) have strong binding affinity. Result of E64 docking shows that strong H-bond interactions formed with backbone oxygen of Leu477and Lys475 and side chain OE2 atom of Glu 586 and nine hydrophobic interactions observed with Met474, Gln479, Cys483, Ala513, Val507, Ile561, His590, Asn591, Leu592, Arg589, Leu581, residues (Table 3). All inhibitors bound to the HEV PCP active site in a manner similar to the inhibitor E64 (Figure 10). In Chymostatin five H-bond interactions formed, two with backbone oxygen of Met474 and Leu477 and one nitrogen atom of Asn591, whereas two side chain H-bond interactions were observed with oxygen atom of Glu479 and Thr482 and eight hydrophobic interactions were observed with Val507, His590, Leu592, Phe576, Glu586, Leu581, Glu583, Leu535 residues. Leupeptin had an H-bond with backbone oxygen of Asn591 and twelve hydrophobic interactions observed with Gln479, Met474, Thr482, Ala513, Val507, Ile517, Ile561, Phe576, His590, Leu592, Arg589, Leu581 residues. Inhibitor ALLN showed two side chain interactions with nitrogen atom of Glu479 and oxygen atom of Glu583, respectively. Eight hydrophobic interactions observed with Val507, His590, Leu592, Arg589, Leu581, Asn591 residues. Among the remaining inhibitors, PMSF Phosparamidon and AEBSF which were found to be inactive in the HEV PCP enzyme inhibition assay, ligand–receptor interaction analysis showed that inhibitor PMSF and Phosparamidon did not formed H-bond interactions (Table 3). Three inhibitors AEBSF, Bestatin and Pepstatin showed week H-bond and hydrohobic interactions. Interestingly, two inhibitors EDTA and Antipain which were inactive in experimental results remain undocked.

**Table 2:** Correlation between relative protease activity of recombinant HEV-PCP and Docking score of the inhibitors.

	**Relative protease activity**	**Docking score**	**Correlation**
Without Inhibitor	100	−	0.75
PMSF	100	−2.6	
E-64	4	−6	
Chymostatin	6	−5.1	
Leupeptin	13	−5.8	
ALLN	11	−4.3	
AEBSF	82	−4.8	
Phosparamidon	95	^*^	
EDTA	77	−	
Antipain	23	−	

* *Although very good docking score −6.8 was found with Phosparamidon but its binding site was different to others and no H-bond interaction were seen (Table 3)*.

**Table 3:** Interaction and distance between the inhibitors and key amino acids of HEV-PCP in XP docking complex determined using LIGPLOT program.

**Inhibitors**	**H-bonds (residue (atom number-distance in Å-ligand atom number) ligand)**	**Other interactions (total hydrophobic interacting residues)**
E64	B-Leu477 (O-3.13-N4)L, B-Leu477 (O-2.91-N5)L, S-Glu586 (OE2-2.60-O3)L, B-Lys475 (O-2.95-N5)L.	(11) Gln479, Cys483, Met474, Ala513, Val507, Ile561, His590, Leu592, Arg589, Leu581, Asn591.
Leupeptin	B-Asn591(N-3.22-O4)L	(12) Gln479, Met474, Thr482, Ala513, Val507, Ile517, Ile561, Phe576, His590, Leu592, Arg589, Leu581.
Chymostatin	S-Thr482 (OG1-3.30-N2) L, B-Met474 (O-2.83-N7) L, B-Leu477 (O-2.89-N7) L, B-Asn591 (N-3.33-O6) L, S-Glu479 (OE1-2.95-N2) L.	(8) Val507, His590, Leu592, Phe576, Glu586, Leu581, Glu583, Leu535.
ALLN	S-Glu479 (NE2-3.01-O2)L, S-Glu583 (OE1-2.84-N2)L.	(8) Val507, His590, Leu592, Arg589, Leu581, Asn591.
PMSF	–	(4) Val507, His590, Leu581, Leu592.
AEBSF	B-Met474(O-2.9-N1)L, B-Leu477(O2-2.92-N1)L.	(5) Gln479, Val507, Leu581, Leu592, Thr482.
Phosphoramidon	–	(10) Lys475, Gln479, Leu477, Leu581, Phe576, Ile561, Leu592, Glu583, Glu588, Arg589.
Bestatin	S-His590(ND1-3.05-O1)L.	(5) Gln479, Leu581, Val507, Asp589, Leu592.
Pepstatin	S-Gln479(OE1-3.14-N4)L, S-Gln479(NE2-2.90-O2)L, S-Glu583(OE1-3.01-N2)L.	(8) Val507, His590, Glu588, Leu582, Leu592, Asp536, Leu535, Phe576

*S, Side chain; B, Backbone*.

## Discussion

Viral proteases are good targets for development of antiviral therapeutics due to their crucial role in viral polyprotein processing. Thus, as a pre-requisite for screening of small molecule inhibitors (SMIs) against HEV protease, we expressed and purified a soluble, catalytically active recombinant viral protease with good yield and homogeneity in baculovirus expression system. Further, we developed a robust cell free assay with high sensitivity, selectivity and reproducibility to screen targeted compounds with anti-HEV protease activity. We validate HEV cysteine protease as a papain like protease on the basis of *in silico* 3D modeling and inhibition with specific inhibitors.

Positive-strand RNA viruses are supposed to encode proteases to process their polyproteins usually required for enzymatic activities, interactions with other proteins, subcellular localization, and the viral assembly (Debing et al., 2016). Processing of HEV ORF1 by its protease domain remains controversial due to unavailability of well-characterized native HEV protease. It was hypothesized that a putative region between residues 433 and 592 of HEV-ORF1 encodes protease (Koonin et al., 1992; Parvez and Khan, 2014) but the nature of protease, its biochemical and biophysical characterization need to be understood. In some reports, role of cysteine protease in HEV processing has been identified but possibility of other proteases could not be ruled out (Ropp et al., 2000; Sehgal et al., 2006).

In previous study HEV protease was expressed from different genotype of HEV in *E. coli* (Accession no: FJ457024 region 440-610) containing extra 9 amino acid (SFDASQSTM) at the C-terminal region have 170 amino acids. This refolded protein was also found to be active and classified as chymotrypsin like protease (Paliwal et al., 2014). In the present study a 161 amino acid long protein of Accession no: AF444002_1 region 432-592 of genotype1, similar to the one predicted by putative domain of HEV (Koonin et al., 1992; Parvez, 2013) has been expressed using baculovirus expression system and purified in native condition. These constructs having significant variation, could result in difference as mentioned by Paliwal et al. (2014) and our study. With the present data it cannot be interpreted the nature of protein with dual properties of papain like Cysteine protease or Chymotrypsin like properties. Further studies will be required to validate this fact. In the present study, recombinant HEV protease was expressed using the baculovirus expression system since it is considered to be an efficient insect expression system with the ability to carry post-translational modifications (Chambers et al., 2018). Previously it has been reported that baculovirus system can express toxic proteins and proteases of different organisms (Keyvani-Amineh et al., 1995; Sali et al., 1998). The cell free assay was developed to quantify the activity of HEV protease since no robust protocols for protease digestion have been described so far. For development of cell free assays, three different approaches have been used to measure protease activity i.e., gelatin zymography, FTC-casein based assay and digestion of full lengthORF1 polyprotein. In pevious report gelatin zymography based assays has been used for hepatitis E viruses (Paliwal et al., 2014), whereby a clearing zone on the slide due to digestion of gelatin was formed. Similar to that, a clear zone was observed on zymogram when the native HEV protease was used. To determine the conditions for the optimal activity of the protease, it has been reported that cysteine protease activates at lower pH (Sundararaj et al., 2012). In our study the protease activity has been shown at lower pH which is typical of a cysteine protease. Digestion of gelatin in zymography gave the first direct evidence of protease activity by the expressed putative protease domain (432–592 aa) of pORF1 leading us to express pORF1 in bacteria to be used as the substrate. The natural activity of HEV-protease could be studied by proteolysis of ORF1 polyprotein which was expressed via *E. coli*. Bl21-DE3 cells. HEV-protease cleaves ORF1 polyprotein in a time dependent manner. After 1 h of incubation, bands of ~100 and ~84 kDa were seen, which further cleaved into smaller products with time and after 5 h, multiple band of smaller size were seen due to proteolysis of ORF1. Digestion of ORF1 by cysteine protease resulted in ~84 kDa RdRp-Hel, and ~67 kDa Met-PCP fragments which were further processed into ~55 kDa RdRp, ~35 kDa Met, and ~27 kDa Hel fragments with higher incubation time. The above obtained fragments were similar in molecular mass to the ones observed in previous immunoprecipitation studies with replicon transfected HepG2 cells (Panda et al., 2000) and after expression of ORF1 polyprotein in cell free system as well as in mammalian cells (Ropp et al., 2000). In addition, ~55 kDa intermediately processed form of the RdRp and~35 kDa Met protein has also been seen in previous study after digestion of 84 kDa RdRp-Hel and 67 kDa Met-PCP fragments by immunoprecipitation (Paliwal et al., 2014). Similar size of products also has been seen in self-digestion of baculovirus derived ORF1 polyprotein (Sehgal et al., 2006). These results suggest role of HEV-protease in ORF1 polyprotein processing and HEV life cycle.

For the first time, enzyme kinetics of native active HEV protease has been determined in this study using FTC-casein substrate based assay to validate enzymatic activity and kinetics of HEV-protease. The values of Km and Vmax were found to be 0.054 mM and 0.77 mM/h, respectively. The optimal temperature for protease activity was found to be 37–48°C which corresponds well to the appropriate environments for HEV growth in the cells (Emerson et al., 2005; Tanaka et al., 2007; Johne et al., 2016). Activity of HEV protease was found to be pH dependent. The protease activity was found to be optimal at low pH of 4 which is the crucial factor to activate Papain like cysteine proteases (Richau et al., 2012; Sundararaj et al., 2012). A number of reports have shown that cysteine proteases can be auto catalytically activated *in vitro* from zymogen to mature enzyme even at reduced pH (Lowther et al., 2009; Sundararaj et al., 2012; Verma et al., 2016) indicating it to be a cysteine protease. In an attempt to validate the nature of the protease we used various inhibitors to monitor its activity. Twelve inhibitors were used to study the inhibition and it was found that the enzyme was completely resistant to the inhibitors of serine proteases (PMSF, AEBSF, aprotinin), aspartic proteases (pepstatin) and metalloproteases (EDTA) while it was moderately inhibited by cysteine protease inhibitor leupeptin, chymostatin and E-64. HEV protease was strongly inhibited by E-64 (99%) and chymostatin (98%), which had been reported to inhibit papain like cysteine protease (Saha et al., 2018). To confirm these results, modeled HEV protease structure has been docked with above inhibitors which shows that cysteine protease inhibitors E64, Chymostatin, Leupeptin, and ALLN have good docking score and strong binding affinity whereas remaining inhibitors PMSF, Phosparamidon, AEBSF, Bestatin and Pepstatin showed no or weak interactions and EDTA and Antipain remain undocked. Correlation coefficient was found 0.75 which showed good agreement between *in vitro* and *in silico* studies, suggesting strongly that the HEV-protease is a Papain like cysteine protease. Besides, *in silico* modeled structure of HEV-protease was found to be similar with Papain like cysteine proteases as it has structurally similar N-terminal helix domain and C-terminal β-sheet domain and conserved Cys–His–Asn triad at the active site (Koonin et al., 1992; Coulombe et al., 1996). Cys483 and His590 form a catalytic triad with Asn 591 which is present in active site between N-terminal helical domain and a C-terminal β-sheet domain of HEV-protease. A histidine residue, presents in the active site act as proton donor and increase the nucleophilicity of the cysteine residue. Nucleophilic cysteine, act up on the carbon of the reactive peptide bond, release of an amine or amino terminus fragment of the substrate and producing the first tetrahedral thioester intermediate (Coulombe et al., 1996). This intermediate is now stabilized by hydrogen bonding between highly conserved glutamine residue and substrate oxyanion. Subsequently, thioester bond is hydrolyzed and form a carboxylic acid moiety from the remaining substrate fragment (Verma et al., 2016). Earlier, the Papain like proteases are characterized by the presence of two domains, an α- helix and a β-sheet domain forming a deep cleft which contains Cys-His-Asn residues along with Gln acting as a substrate-binding site (Turk et al., 2001). Similar observation has been recorded in our predicted 3D model showing two domain architecture structures where the Cys-His-and Asn triad is formed along with Gln at the active site. Further, according to our findings, the Cys483 and His590 forms a catalytic triad with Asn 591 present in active site between N-terminal helical domain and a C-terminal β-sheet domain of HEV-protease. Further, our findings are also in agreement with previous study where HEV-protease has been categorized as a Papain like cysteine protease (Koonin et al., 1992; Parvez and Khan, 2014). In this study HEV protease was found to have these characteristics and on the basis of structural similarity with papain like protease it has been characterized as papain like cysteine protease. These findings are in the agreement of previous study where HEV-protease has been categorized as a Papain like cysteine protease (Koonin et al., 1992; Parvez and Khan, 2014).

Effect of different divalent cations like Ca^2+^, Cu^2+^, Mg^2+^, Mn^2+^, Ni^2+^, Zn^2+^, Fe^2+^, and Pb^2+^ were studied on activity of HEV protease. Out of these divalent cations Zn^2+^ remarkably decreased protease activity. Divalent cations like zinc and its conjugates have been identified as protease inhibitors for SARS-CoV proteases (Hsu et al., 2004), also zinc have been found to be essential for Structural stability, folding of NS3 protease of HCV (Urbani et al., 1998) and catalytic activity of Rubella protease (Liu et al., 1998). In another report, Zn has been found to be an essential structural cofactor for human corona virus papain like protease (Herold et al., 1999). Thus, we were interested in testing the influence of metal conjugated compound on HEV protease activity. Further, *in silico* studies have been performed to determine the zinc binding sites present in HEV protease. Two zinc binding sites have been identified, as “Zn^2+^ binding motif” Cys457-His458-Cys459 (CHC) and Cys481-Thr482-Cys483 (CTC) and these findings are in agreement with previous report (Parvez and Khan, 2014) who studied that mutation in Cys 481 and Cys 483 leads to loss of protease activity (Parvez, 2013; Paliwal et al., 2014). These residues present in the zinc binding site may play role in its structural stability and proper folding. It was reported that zinc binding site mediates proteolytic activity of Corona viral papain like protease (Berg and Shi, 1996). These findings support our report where the loss of protease activity has been found in the presence of zinc, which may act as a cofactor and confer the structural stability.

Most of the RNA viral proteases are categorized either as chymotrypsin like or papain-like proteases (Neurath, 1984). Chymotrypsin like proteases have been found to exhibited with three different catalytic triads Ser-His-Asp, Cys-His-Asp, and Cys- His-Glu (Bazan and Fletterick, 1988; Gorbalenya et al., 1989; Matthews et al., 1994), whereas papain like proteases are found to have the conserved Cys-His dyad which are assisted by unique Asn and Asp residues (Gorbalenya et al., 1991). In both the chymotrypsin-like or papain-like proteases, Zn^2+^ is located at the site opposite to the active center (Herold et al., 1999). Therefore, the classification of HEV protease not only depends on the involvement of cysteine and histidine but also on structure and sequence homology. Papain like cysteine proteases are very stable enzymes and often are found in proteolytically harsh environments (Richau et al., 2012). Besides catalytic dyad/triad prediction we find that HEV-protease activity was found to be optimal at low pH of 4 which is the crucial factor to activate cysteine proteases. It is already reported that cysteine protease activates at lower pH (Sundararaj et al., 2012). In our study the protease activity has been shown at lower pH which is typical of a cysteine protease. On the basis of structural homology, active site residue and class specific inhibition we have classified HEV-protease as a “Papain-like cysteine protease.”

## Materials and Methods

### Cells and Virus

*Spodoptera frugiperda* (Sf21) cells (Invitrogen, California, US) were used for generation, propagation and titration of recombinant baculovirus. Sf21 cells were maintained in Sf900III insect cell medium (Gibco, Newyork, US) and supplemented with 10% fetal bovine serum (Gibco, Newyork, US) at 27°C in refrigerated incubator. HEV cDNA clone pSK-HEV-2 genotype 1 GenBank accession no. AF444002 was used as a reference virus in this study. All the virus experiments were performed in BSL-2 facility in Shiv Nadar University, Dadri, India.

### Cloning of HEV Protease Using Bac to Bac Expression System

The HEV protease domain (nts 1319–1801t of HEV; GenBank accession no. AF444002) was PCR-amplified by 5′CTCAGAATTCATGGCTCAGTGTAGGCGCTG3′ and 5′TCTAGCGGCCGC GAGATTGTGGCGCTC6TGG3′ forward and reverse primer from full length HEV cDNA clone pSK-HEV-2 using Pfu DNA polymerase (NEB, Massachusetts, US). Amplified product was cloned into pFastBac/CT-TOPO vector (Invitrogen, California, US) following manufacture's protocol and transformed into One Shot™ Mach1™ T1R Chemically Competent *E. coli* (Invitrogen, California, US). Positive transformants were screened by PCR and confirmed by DNA sequencing. Further, pFastBac/CT-HEV protease construct was transformed into MAX Efficiency™ DH10Bac™ Competent *E. coli* (Invitrogen, California, US) that contains baculovirus shuttle vector or bacmid. Positive Colonies were confirmed by PCR amplification using pUC/M13 reverse and forward primers. The recombinant bacmid was purified using PureLink HiPure Plasmid Miniprep Kit (Invitrogen, California, US) following manufacture's protocol.

### Generation of Recombinant Baculovirus Containing HEV-Protease

Recombinant bacmid containing HEV protease gene was transfected in Sf21 cells using Cellfectin™ II reagent (Invitrogen, California, US). Briefly 8 × 10^5^ Sf21 cells /well were seeded in six well plates. Five-hundred nanogram bacmid DNA was mixed with cellfectinII reagent and transfected into Sf21 cells. After 1 h incubation at 27°C, complete Sf900III medium was added and plate incubated at 27°C for 72 h. Supernatant was collected and titrated using plaque assay. Further, plaques were purified and amplified in Sf21 cells while the recombinant baculovirus was confirmed through PCR of genomic DNA of the infected Sf21 cells.

### Demonstration of HEV Protease Expression in Sf21 Cells by Immunofluorescence Assay

In order to demonstrate the expression of HEV-protease in Sf21 cells, 1 × 10^6^ Sf21 cells were seeded in six well plates and infected at 1 MOI (multiplicity of infection) with recombinant baculovirus clone having HEV-protease gene. After 48 h of infection, cells were fixed with chilled methanol for 30 min, followed by permeabilisation with 0.1% Triton X-100 for 15 min. The cells were blocked for 1 h at room temperature with 3% BSA in PBST (PBS with 0.1% Triton X100). Infected Sf21 cells were probed with HEV-protease epitope specific antibodies constructed by Genscript against 444–457 peptide region of HEV-protease (Table 1) for 1 h at room temperature. After three washing with PBS, the cells were stained with goat anti-rabbit IgG-Alexa Fluor 488 (Biogenix) for 60 min at room temperature. Subsequently, the cells were washed with PBS and their nuclei were stained with DAPI (4′,6-diamidino-2-phenylindole) (Sigma) for 10 min at room temperature. The cells were washed and fluorescence was visualized using a fluorescence microscope (Olympus IX 71, Germany).

### Expression of Recombinant HEV-Protease in Sf21 Insect Cells

Expression of HEV-protease was performed in Sf21 cells as described previously (Sehgal et al., 2006). Briefly 2 × 10^7^ Sf21 cells were seeded in T175 tissue culture flask and incubated at 27°C to attach the cells. Cells were washed with incomplete SF900 III medium and infected with the recombinant baculovirus at MOI of 10. The cells were incubated at 27°C for 90 min followed by addition of 20 ml SF900III medium (Invitrogen, California, US) containing 10% FBS and incubated at 27°C for 48 h. The infected Sf21 cells were daily observed for cytopathic effect (CPE) and harvested after 48 h of infection, when the CPE was ~95%. The harvested cells were centrifuged at 1,500 × g for 10 min at 4°C, washed using PBS and stored at −80°C until further use.

### Solubilization and Purification of Recombinant HEV-Protease

Extraction of HEV-protease from Sf21 cells was performed, as described earlier (Ramya et al., 2011) with some modifications. In order to solubilise the HEV-protease, 2 × 10^7^ infected Sf21cells were resuspended in 1 ml resuspension buffer (50 mM Tris-HCl pH 8.0, 150 mM NaCl, 10% DMSO and 1% CHAPS) and incubated on ice for 30 min. The cells were sonicated for 2 min (2 s on 30 s off) at 20% amplitude on ice and incubated for 4 h at 4°C in end to end rotator. Cell lysate was centrifuged at 21,000 x g for 1 h at 4°C. Solubilized protein was purified through immobilized metal affinity chromatography (IMAC) using Ni–NTA super flow chelating agarose column (Qiagen, Germany). The column was equilibrated with buffer A (50 mM Tris-HCl, 150 mM NaCl, 0.5% CHAPS and 10 mM Imidazole pH 8.0), followed by washing with buffer B (50 mM Tris-HCl, 150 mM NaCl, 0.5% CHAPS and 20 mM Imidazole pH 8.0). The protein fraction was eluted in buffer C (50 mM Tris-HCl, 150 mM NaCl, 0.5% CHAPS and 250 mM Imidazole pH 8.0) and analyzed on 12% SDS-PAGE. Theelutes were pooled and dialyzed against PBS and analyzed by Western blot using HEV-protease epitope specific antibodies (Genscript, US) (Table 1).

### Determination of Protease Activity by Gelatin Zymography Assay

Activity of purified recombinant HEV-protease was determined by gelatin zymography, as described earlier (Saitoh et al., 2007). Briefly 12% SDS-PAGE having 4 mg/ml gelatin was polymerized in 1 mM thickness plate. Different quantities of recombinant HEV-protease (1–10 μg), cell lysate of uninfected cells and trypsin were loaded as negative and positive control, respectively. The gel was run at 100 Volt until separation, washed twice for 30 min at 37°C with wash buffer (2.5% Triton X-100, 50 mM Tris-HCl, pH 7.5, 5 mM CaCl_2_ and 1 μM ZnCl_2_) to remove SDS. The gel was rinsed twice with distilled water and incubated at 37°C in incubation buffer (1% Triton x 100, 50 mM Tris-HCl, pH 6.8, 5 mM CaCl_2_ and 1 μM ZnCl_2_) for 24 h. After incubation, gel was stained by coomassie brilliant blue R (CBBR) (0.5 g in 40% methanol, 10% acetic acid, and 50% distilled water). A clear zone was visualized after destaining with 40% methanol, 10% acetic acid, and 50% distilled water.

### ORF1 Polyprotein Digestion Assay

The role of HEV-protease in ORF1 polyprotein processing was determined by digestion assay. Briefly, full length ORF1 polyprotein was expressed in *E. coli*. BL21 cells and purified by Ni-NTA chromatography and characterized by Western blotting using HEV epitope specific antibodies (Table 1). The 186 kDa protein was used as a substrate for HEV-protease. To set up digestion assay, 100 ng of ORF1 polyprotein was incubated with 1 ng of HEV-protease in 0.1 M Tris-HCl (pH 7.2) and incubated at 37°C for 1–5 h. Further, to check internal protease activity in full length ORF1, it was incubated without HEV-protease for 24 h. After incubation, protein was fractioned on SDS-PAGE and size of digested products was checked by Western blotting using epitope specific antibodies against all four enzymes present on ORF1 viz. Methyl transferase, Cysteine protease, Helicase and RdRP (Table 1).

### Fluorescein Thiocarbamoyl-Casein Based Protease Assay

FTC-casein based assay was performed to check the activity of protease as described earlier (Twining, 1984). Fifty micrometer FTC-casein was incubated with 1 μM HEV-protease for 1–12 h at 37°C. Similar reaction was set up with trypsin as a positive control and without any protease. Protease cleaves FTC- casein into smaller, TCA-soluble, FTC-peptides. 0.5% TCA was added to the reaction mixture, to precipitate any remaining undigested FTC-casein. The supernatant was collected following centrifugation at 12,000 g at 4°C and the FTC-peptides were quantified by measuring the absorbance at 492 nm in spectrophotometer. The intensity of color estimated by the assay was found to be proportional to the total protease activity present in the sample.

### Biochemical Characterization of HEV-Protease

Biochemical properties of HEV-protease were characterized through kinetic studies under varying reaction conditions like pH (4–12), temperature (4–60°C), salt concentration (0−300 mM NaCl), presence of glycerol (0–50%) and various enzyme and substrate concentration described previously (Ahmed et al., 2011). Finally apparent kinetic parameters (V_max_ and K_m_) of the HEV-protease were determined by FTC-casein based protease assay. The initial velocity was measured using varying substrate concentration and the reciprocal of initial velocity and substrate concentration were plotted using Lineweaver-Burk curve to determine Briefly, Different concentration of the substrate (0.001–1.2 mM) was incubated with 1 μM HEV-protease in 4X incubation buffer (200 mM Tris-HCl, pH 7.8, 20 mM CaCl_2_) at 37°C. To measure enzyme kinetics, absorbance was taken in multiplate reader (Bio-rad) at 492 nm for 1–12 h and initial velocity of each reaction was determined. Finally, the reaction rate was calculated using Michaelis-Menten equation (Michaelis and Menten, 1913)

$$\begin{array}{l} \text{V}=\text{Vmax }\left[\text{S}\right]/\text{Km}+\left[\text{S}\right] \end{array}$$

Vmax is the maximum rate of reaction and Km is the substrate concentration at which reaction rate is half of the obtained Vmax.

Further, the effect of various activators or divalent cations on the protease activity was examined by supplementing the reaction with 20 mM of CuCl_2_, NiCl_2_, MgCl_2_, CaCl_2_, ZnCl_2_, MnCl_2_, FeCl_2_, and PbCl_2_ as described above. Similar reaction was also performed in the presence of various types of protease inhibitors (Protease inhibitor set; G Biosciences) to determine the nature of protease by inhibition assay, Briefly, 100 μl (1 μM) HEV protease was pre-incubated with 1× protease inhibitors AEBSF, ALLN, antipain dihydro chloride, aprotonin, bestatin, chymostatin, sodium EDTA, E-64, leupeptin, pepstatin, phosphoramidon and PMSF and the inhibition assay was performed using FTC-casein. Inhibition of protease activity was measured as relative absorbance at 492 nm. For relative inhibition activity, same reaction was performed without any inhibitors. In order to validate the inhibition, ORF1 digestion was performed in presence of these inhibitors. A protease-negative control with 100 ng pORF1 was incubated in parallel under the same conditions to monitor pORF1 autolysis. These reactions were incubated for 5 h at 37°C for over-digestion. All the reactions and controls were resolved by 10% SDS-PAGE.

### *In silico* Studies of HEV Protease

The 3D model of HEV PCP (AAL50055.1, region 432–592) was generated using homology modeling, fold recognition and threading techniques (Zhang, 2008; Roy et al., 2010; Yang et al., 2015). Initially I-TASSER was used for modeling of 161 amino acid residues; unfortunately only C-Terminal region could be modeled using 6NU9 as a template and N-Terminal region remain unmodeled. Integrated Protein Structure and Function Prediction Server IntFOLD Version 5.0 (Roche et al., 2011; McGuffin et al., 2015, 2019) was used for modeling of N-Terminal residues 432–513 Amino Acids of ORF1. Finally easy modeler was used for final model generation (Kuntal et al., 2010). The generated model was energy minimized in water using OPLS (optimized potencials for liquid simulations) force field with the convergence threshold of 0.05 by using Macromodel of Maestro (MacroModel, 2017), to remove steric clashes between atoms and to improve overall structural quality of predicted models (Sastry et al., 2013; Protein Preparation Wizard, 2017). 3D model was validated on the basis of stereochemical and geometric consideration and docking studies (Glide, 2017). The top ranked model was further validated and analyzed based on their Ramachandran plot, Verify 3D and ERRAT analysis (Bowie et al., 1991; Lüthy et al., 1992; Laskowski et al., 1993; Colovos and Yeates, 1993). To identify possible binding sites, site map of maestro was used for binding site prediction (Halgren, 2007, 2009; SiteMap, 2017). Further, Receptor grid generation was done using Glide module. Docking study was done in using Grid based ligand docking with energetic (GLIDE) (Friesner et al., 2004, 2006; Halgren et al., 2004). The 3D model of HEV PCP was docked with inhibitors retrieved from Pubchem search. Eleven known protease inhibitors were docked against HEV-PCP structural model. The different conformations of the compounds were docked flexibly and maximum 1,000 poses per compound were generated (Ligprep, 2017). The analysis of the poses, complexes and the binding affinities between the receptor and ligands was analyzed using Schrodinger's software Glide and correlation coefficient between inhibitory concentration and docking score was calculated using “CORREL” function of MS Excel (Microsoft corp., USA).

### Statistical Analysis

For enzyme kinetics, initial velocities were calculated using the linear regression function in the Microsoft Office excel software. Data was analyzed and plotted using Michaelis-Menten equation with Graph Pad Prism to obtain kinetic parameters. All the assays were performed in triplicate and results were graphed, with error bars indicating the SD. Inhibition assay data was analyzed using student's *t*-test *p* < 0.05 was considered statistically significant. Each experiment was performed thrice in triplicate.

## Data Availability Statement

All datasets generated for this study are included in the article/Supplementary Material.

## Author Contributions

DS conceived the study, designed the experiments and prepared the manuscript. SS designed, carried out the experiments performed the data analysis and drafted the manuscript. MC designed, carried out the insilco work and data analysis. All the authors reviewed the manuscript.

### Conflict of Interest

The authors declare that the research was conducted in the absence of any commercial or financial relationships that could be construed as a potential conflict of interest.
