# Supplementary material for: Hepatitis E Virus Cysteine Protease Has Papain Like Properties Validated by in silico Modeling and Cell-Free Inhibition Assays
Source: Front Cell Infect Microbiol. 2020 Jan 23;9:478. doi: 10.3389/fcimb.2019.00478 (PMC6989534; doi:10.3389/fcimb.2019.00478)
Supplement: Supplementary file 1 [file Data_Sheet_1.pdf]

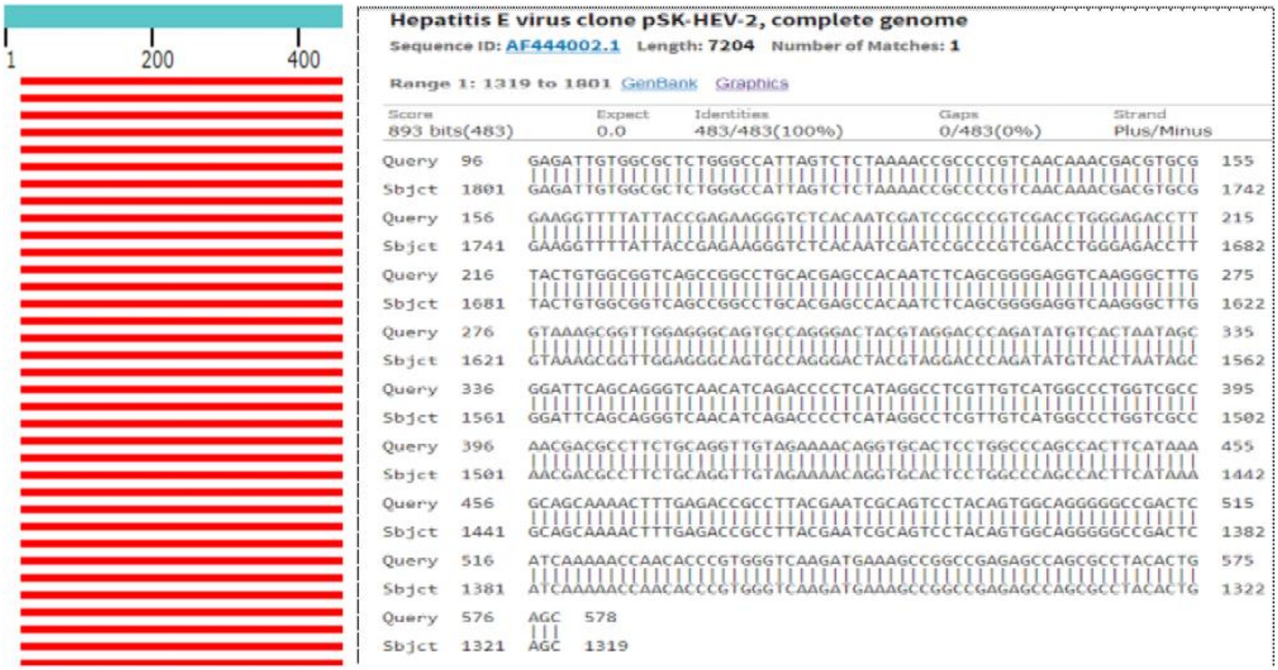

Supplementary fig 1: Sequencing data after NCBI blast . Alignment showing similarity with HEV protease region of genotype 1.

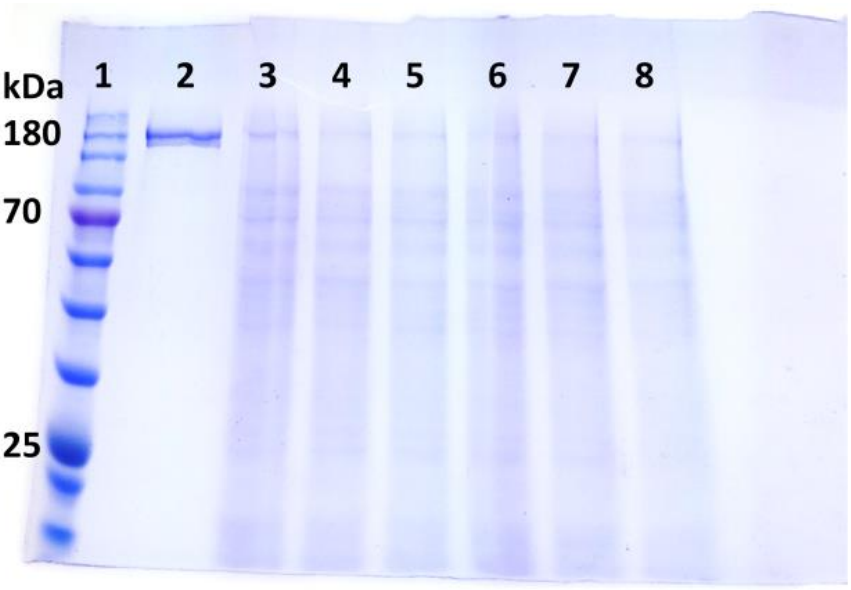

Supplementary fig 2: SDS PAGE and coomassie blue staining after digestion of ORF1 with HEV protease at different time points Lane 1 Marker, Lane 2 undigested ORF1, Lane 3-8 digested ORF1 at 1,2, 5, 8,10 and 12 hrs post digestion.

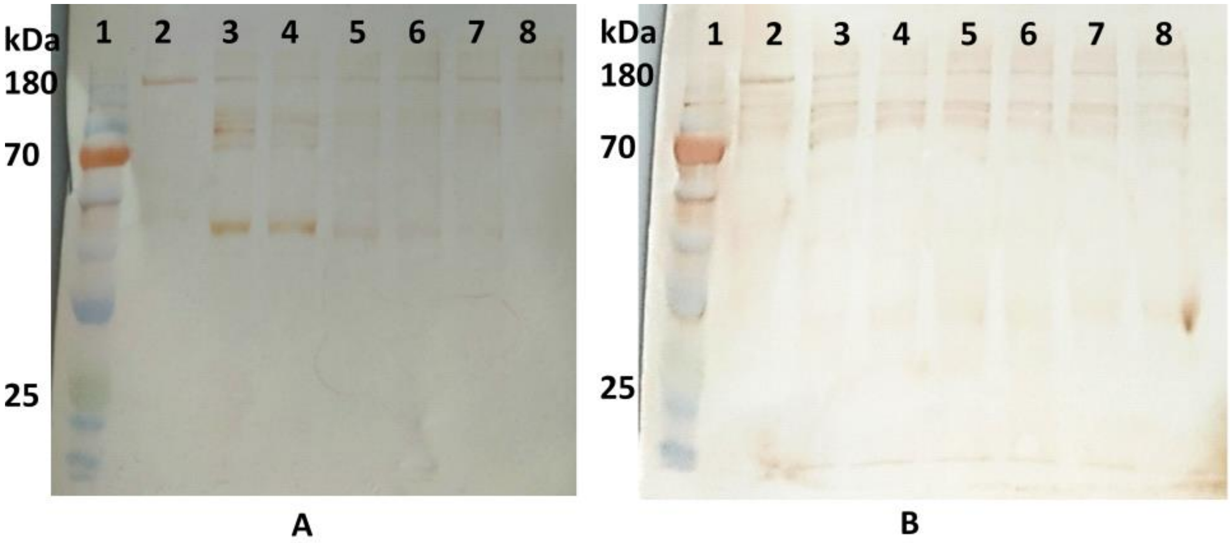

Supplementary fig. 3: Panel A and B represents western blot analysis using anti RdRp and anti Hel antibodies after digestion of ORF1 with HEV protease at different time points Lane 1 Marker, Lane 2 undigested ORF1, Lane 3-8 digested ORF1 at 1,2, 5, 8,10 and 12 hrs post digestion.

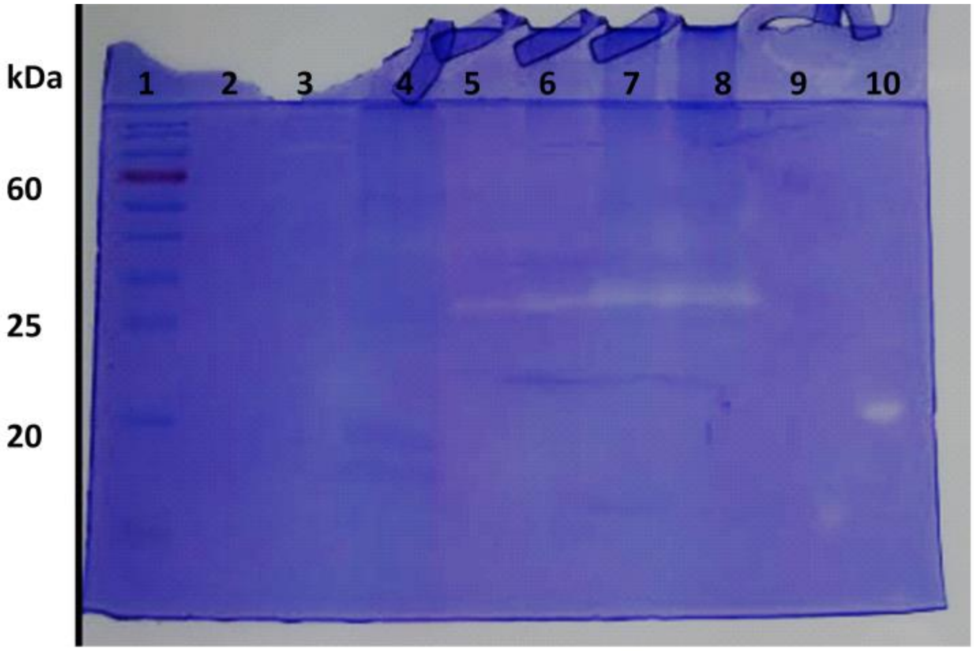

Supplementary fig 4: Zymography of HEV protease using gelatin as a substrate. Lane M, protein ladder, Lanes 1 and 2 were loaded with 10 ul and 20 µl of solubilization buffer to eliminate any contaminating protease activity, Lane 3 was loaded with 20 ul cell sup of uninfected Sf21 cells, Lane 4-7 were loaded with increased quantity of HEV-protease (5,10,15 and 20 ng, respectively), Lane 8 was left blank, and Lane 9 was loaded with 5 ng of trypsin as a positive control.
