# Supplementary figures and images for: Hepatitis E Virus Cysteine Protease Has Papain Like Properties Validated by in silico Modeling and Cell-Free Inhibition Assays
Source: Front Cell Infect Microbiol. 2020 Jan 23;9:478. doi: 10.3389/fcimb.2019.00478 (PMC6989534; doi:10.3389/fcimb.2019.00478)

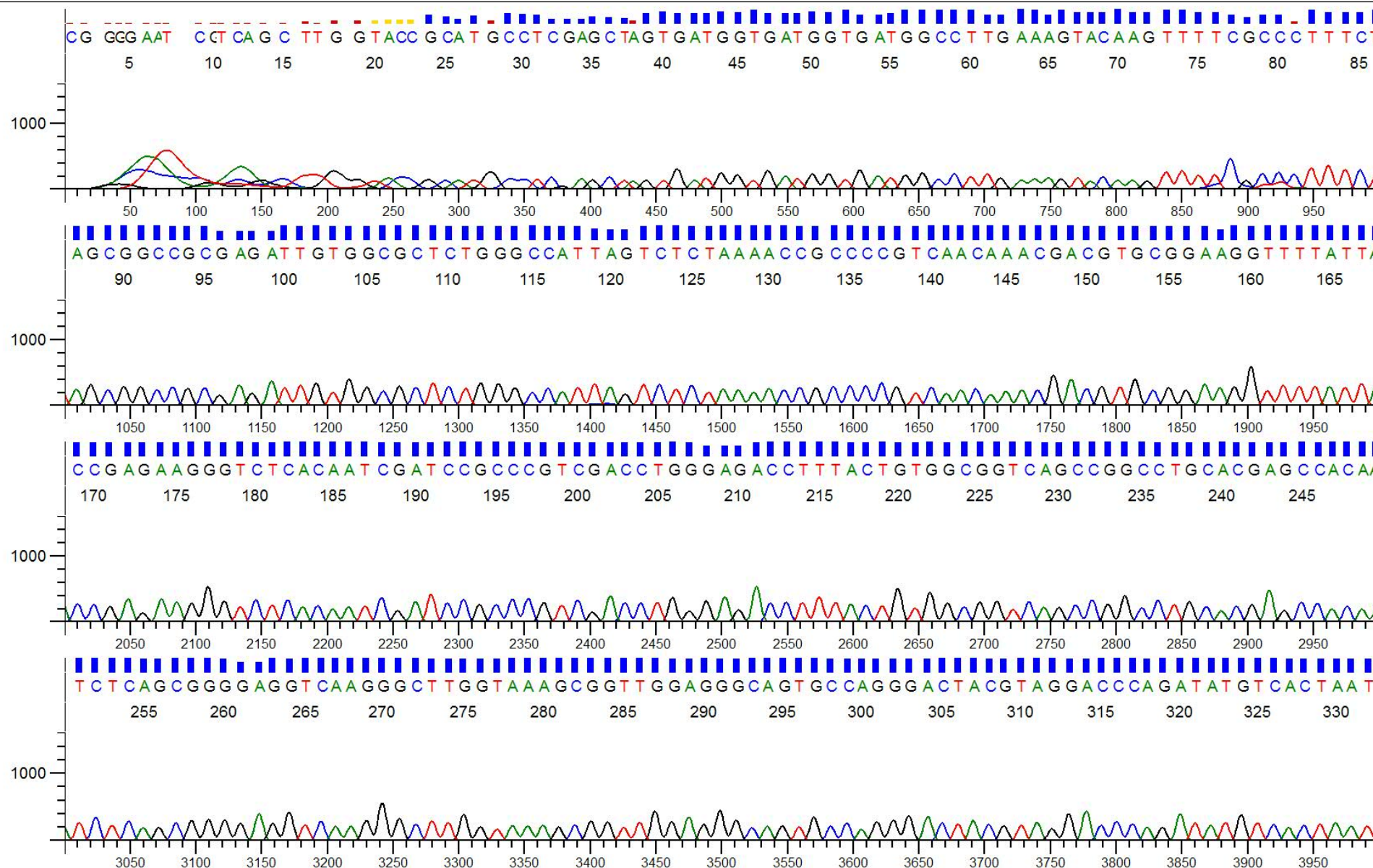

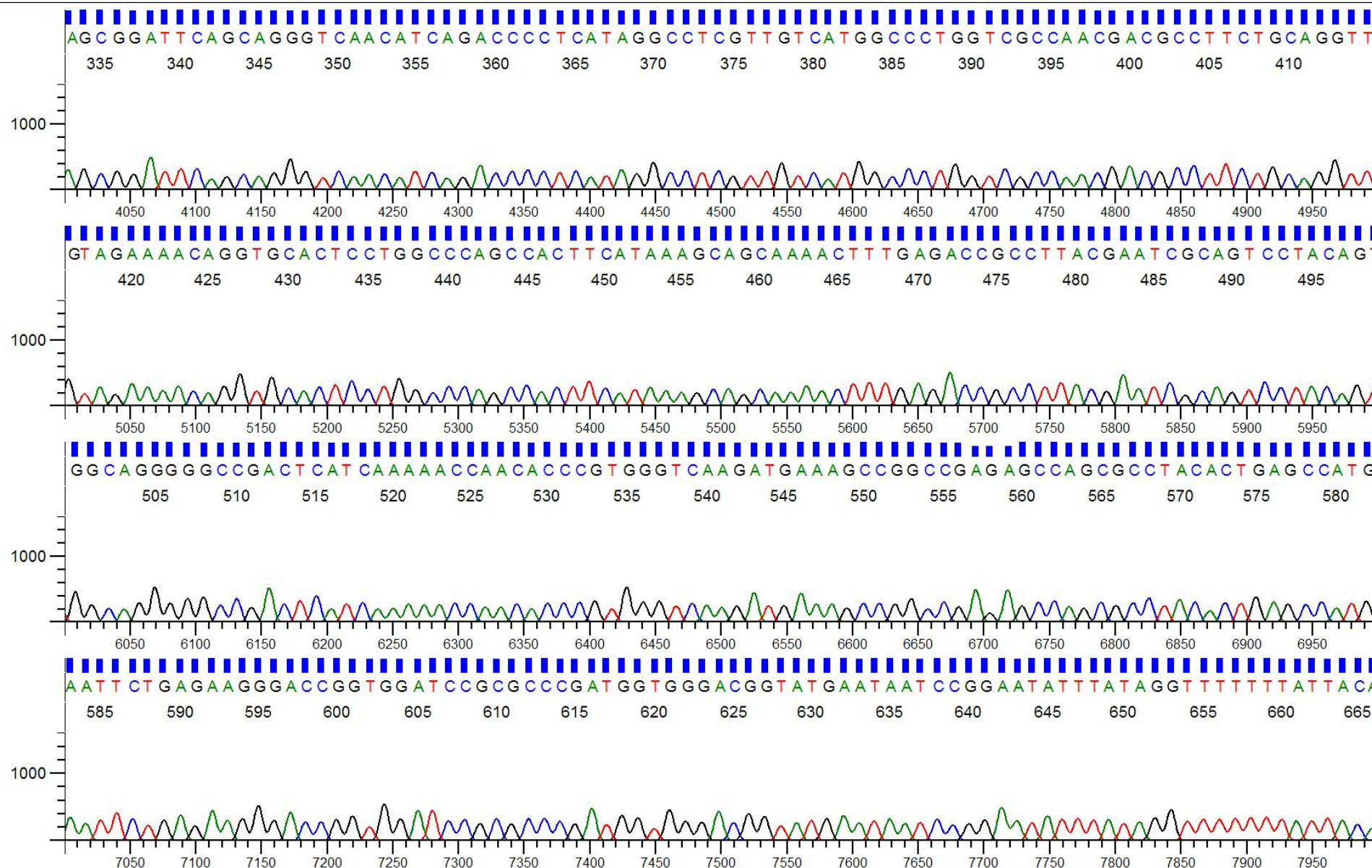

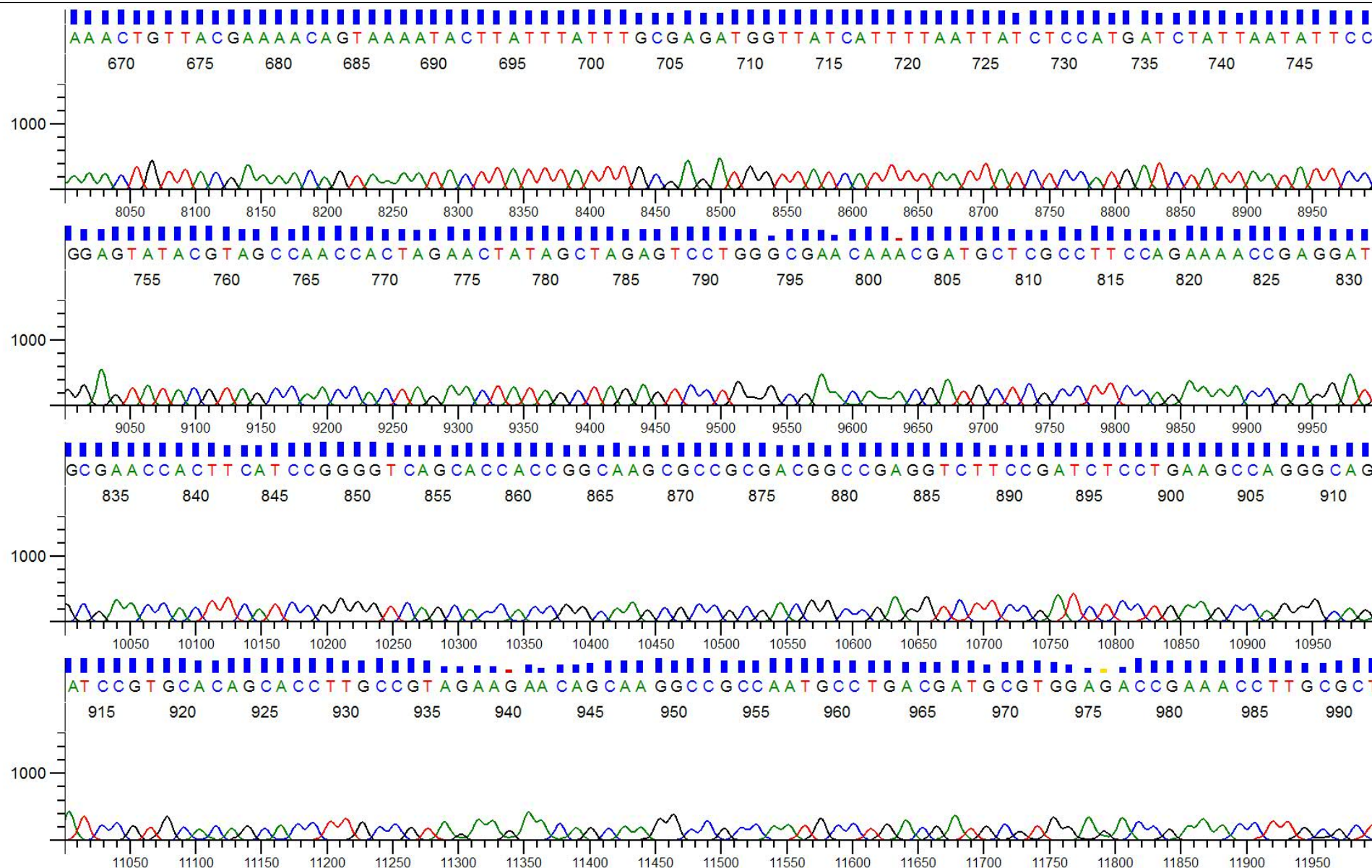

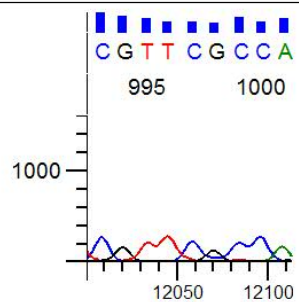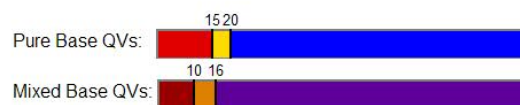

Supplement: Supplementary file 2 [file Data_Sheet_2.PDF]
